# Immune response in blood before and after epileptic and psychogenic non-epileptic seizures

**DOI:** 10.1016/j.heliyon.2023.e13938

**Published:** 2023-02-21

**Authors:** Matilda Ahl, Marie K. Taylor, Una Avdic, Anna Lundin, My Andersson, Åsa Amandusson, Eva Kumlien, Maria Compagno Strandberg, Christine T. Ekdahl

**Affiliations:** a Division of Clinical Neurophysiology and Department of Clinical Sciences, Lund University, Sweden; b Lund Epilepsy Center, Department of Clinical Sciences, Lund University, Sweden; c Clinical Neurophysiology, Department of Medical Sciences, Uppsala University, Uppsala, Sweden; d Department of Medical Sciences, Uppsala University, Uppsala, Sweden; e Division of Neurology, Department of Clinical Sciences, Lund University, Sweden

**Keywords:** Epilepsy, Psychogenic non-epileptic seizure, EEG, Immune response, Biomarker, Interleukin-6

## Abstract

**Inflammatory processes** may provoke epileptic seizures and seizures may promote an immune reaction. Hence, the systemic immune reaction is a tempting diagnostic and prognostic marker in epilepsy. We explored the immune response before and after epileptic and psychogenic non-epileptic seizures (PNES). Serum samples collected from patients with videoEEG-verified temporal or frontal lobe epilepsy (TLE or FLE) or TLE + PNES showed increased interleukin-6 (IL-6) levels in between seizures (interictally), compared to controls. Patients with PNES had no increase in IL-6. The IL-6 levels increased transiently even further within hours after a seizure (postictally) in TLE but not in FLE patients. The postictal to interictal ratio of additionally five immune factors were also increased in TLE patients only. We conclude that immune factors have the potential to be future biomarkers for epileptic seizures and that the heterogeneity among different epileptic and non-epileptic seizures may be disclosed in peripheral blood sampling independent of co-morbidities.

## Highlights

• Elevated IL-6 levels in serum in patients with temporal and frontal lobe epilepsy
• Altered postictal-interictal ratio of immune factors in serum after temporal but not frontal lobe seizures
• No change in IL-6 levels in serum in patients with psychogenic non-epileptic seizures

## 1 Introduction

An increasing body of evidence from experimental studies indicates not only local but also systemic immune reactions as hallmarks of epilepsy [1,2]. A bidirectional interaction seems to exist where **inflammatory processes** may provoke epileptic seizures and seizures may promote an immune reaction [[3], [4], [5]]. **Immune factors such as cytokines and chemokines are known to induce synaptic modulation and contribute to neuronal hyperexcitability** [[6], [7], [8]]. Hence, the systemic immune reaction is a tempting biomarker in epilepsy for both diagnostic and prognostic purposes. However, previous studies on blood from patients with epilepsy are not presenting uniform but rather contradictory pictures. The pro-inflammatory cytokine interleukin-6 (IL-6) has been found to be increased in cerebrospinal fluid and serum after severe generalized tonic clonic seizures in adult patients with epilepsy [9,10]. Increased interictal (time between seizures) serum levels of IL-6 together with IL-1b, IL-1Ra, IL-8, INFy, IFNlambda3 and IL-17 have also been correlated to seizure severity in patients with different types of epileptic seizures [11]. Other studies have reported increased interictal levels of IL-6 in temporal lobe epilepsy (TLE) but not in extra-temporal lobe epilepsy (XTLE) or increase of IL-6, IL-17 and interferon-y (IFN-y) in TLE but only IL-17 and IFN-y in XTLE [12,13]. On the contrary, some studies reject increased interictal levels of IL-6, IL-1B, and IL-1Ra in epilepsy patients, and report only postictal increase (within 24 h after a seizure/ictal event) in IL-6 after TLE, XTLE, and focal epilepsy, or postictal increase in IL-6 in TLE and not in XTLE patients [12,[14], [15], [16], [17], [18]].

Interictal and postictal serum levels of immune factors are challenging to standardize due to the unpredictable nature of epileptic seizures and uncertainties in self-reported seizures as a result of blurred awareness and recall during or after a seizure. This is particularly true for non-convulsive seizures without prominent motor symptoms. Consequently, individual immune factors may fluctuate over time (hours-days) in the same patient depending on e.g. seizure frequency and time since last seizure, which generates significant variations in the so called chronic levels. Moreover, the seizure etiology (i.e. lesions, infections, genetics) may confound results, as do comorbidities and psychogenic non-epileptic seizures (PNES), the latter often difficult to distinguish from epileptic seizures. Collectively, this emphasizes the need for video-electroencephalogram (vEEG) -verified interictal, ictal and postictal time periods when evaluating seizure-induced immune responses in research reports.

In the present study, we explore a systemic immune reaction in blood both before and after 2 different types of epileptic seizures (temporal and frontal lobe seizures) and PNES in a cohort of patients undergoing simultaneous continuous vEEG recordings. We correlate 25 serum protein levels to several clinical parameters such as seizure burden, epileptiform activity on EEG, MRI brain pathology, co-morbidities, physical training and anti-seizure medications (ASMs).

## 2 Results

### 2.1 Patient inclusion and clinical characteristics

**Fig. 1:**
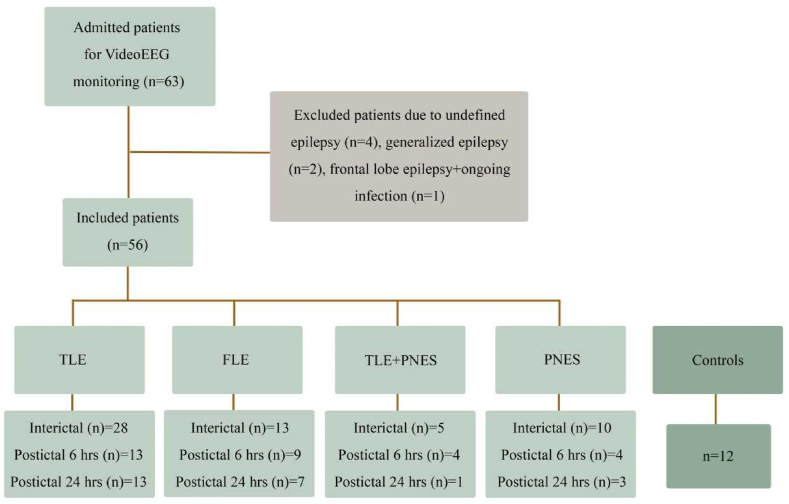
Study flow chart indicating the enrolment of patients with temporal and frontal lobe epilepsy (TLE and FLE) and psychogenic non-epileptic seizures (PNES) for blood sampling on hospital admission (interictal) and after video-EEG verified seizures (postictal).

In total, 63 patients admitted to either Skane University Hospital (n = 56) or Uppsala University Hospital (n = 7) for three to five days of vEEG monitoring and 12 control subjects were included in the study (Fig. 1). Seven patients were excluded due to undefined epilepsy diagnosis (n = 4), generalized epilepsy (n = 2; too few for statistical analyses) or concomitant infection (n = 1). The remaining 56 patients were divided into four groups based on observed seizure semiology and EEG findings: TLE (n = 28), frontal lobe epilepsy (FLE) (n = 13), TLE + PNES (n = 5) and PNES (n = 10).

Demography, medical history, and self-reported seizure characteristics of patients on admission to vEEG monitoring are presented in Table 1. The age ranged from 18 to 64 years, with more women than men in all groups except the FLE group, where 10 out of 13 were men, corroborating the known male dominance in FLE patients [19]. However, there were no gender differences between the individual patient groups and the control (CTRL) group (TLE vs CTRL p = 0.61, FLE vs CTRL p = 0.11, PNES vs CTRL p = 0.12 and PNES + TLE vs CTRL p = 0.13). The median number of current ASMs was the same for TLE and FLE patients (n = 2) and higher in the FLE compared to the PNES group (p = 0.022). Six out of 10 patients with only PNES had 1-2 ASMs. At least 5 of them had their medication withdrawn after the vEEG monitoring. Patients with TLE, FLE and PNES had higher prevalence of self-reported comorbidity compared to controls (p = 0.002, 0.032, 0.003, respectively). MRI findings were common in all patient groups without subgroup differences. Patient-reported sleep disturbances were relatively infrequent. Amount of self-reported physical activity was not different between groups.

**Table 1:** Demography, medical history and reported seizure characteristics in patients on hospital admission for continuous videoEEG monitoring.

Variable	TLE (28)	FLE (13)	TLE with PNES (5)	PNES (10)	Control (12)	P-value
**Demography**						
**Age: median years (range)**	31 (19–63)	25.5 (20–51)	38 (36–63)	34.0 (18–64)	31.5 (22–50)	0.48^2^
**Gender: women % (n)**	57 (16)	21 (3)	100 (5)	90 (9)	58 (7)	0.005^1^
**Medical history**						
**Current ASMs: median no (IQR)**	2.0 (1.0–2.75)	2.0 (1.0–3.0)	2.0 (1.5–3.0)	1.0 (0.0–1.25)	–	0.008^2^
**Reported co-morbidities: % (n)** **-psychiatric** **-neuropsychiatric** **-miscellaneous**	39 (11)	15 (2)	20 (1)	60 (6)	0 (0)	0.004^1^
	-n = 9	-n = 2	-n = 0	-n = 5		
	-n = 1	-n = 1	-n = 0	-n = 2		
	-n = 2	-n = 0	-n = 1	-n = 2		
**MRI findings: % (n)**	57 (16)	54 (7)	50 (3)	20 (2)	–	0.21^1^
**Reported sleep disturbances: % (n)**	14 (4)	7 (1)	0 (0)	10 (1)	0 (0)	0.86^1^
**Reported physical activity grade 1**–**3: median (IQR)**	1 (0–1.75)	0 (0–1)	0 (0–1)	1 (0–2)	1 (0–2)	0.48^2^
**Reported seizure characteristics on admission**						
**Seizure semiology last 6m incl. bilateral convulsive % (n)**	Fo_IA_NM = 25 (7)	Fo_A = 8 (1)	A = 20 (1)	A = 30 (3)	–	–
	Fo_IA_M = 4 (1)	Fo_A_Mtonic = 8 (1)	HM = 60 (3)			
	Fo_IA_Mauto = 18 (5)	Fo_A_Mhyperkinetic = 8 (1)				
	Fo_IA_BC = 46 (13)	Fo_IA_NM = 8 (1)		HM = 40 (4)		
	Fo_IA = 4 (1)	Fo_IA_M = 23 (3)				
	Fo_M = 4 (1)	Fo_IA_Mtonic = 15 (2)	SS = 20 (1)			
		Fo_M = 8 (1)		SS = 20 (2)		
		Fo_Mhyperkinetic = 15 (2)				
		Fo_Mtonic = 8 (1)				
**Seizure frequency last 6m: median no/week (IQR)**	0.75 (0.25–2.0)	5.5 (0.88–11)	4 (2.1–5)	1.5 (0.25–10.5)	–	0.022^2^
**Time since last seizure: median days (IQR)**	7 (7–30)	7, 1-7	7, 1-30	7, 1-30	–	0.21^2^
**Seizures during night time: % (n)**	55 (12)	82 (9)	20 (1)	25 (2)	–	0.038^1^

On admission to the hospital, patient-reported seizure semiology included a variety of stereotypic behaviour and seizure frequency ranging from 0.25 to 11 times/week, both night and day, but with more nocturnal seizures in FLE compared to the PNES and PNES + TLE groups (p = 0.024 and 0.036, respectively). TLE patients frequently reported impaired awareness while the FLE patients reported predominantly hyperkinetic and tonic movements. PNES patients described subjective and akinetic as well as hypermotor symptoms. Time since last seizure on admission to hospital was on average one week for all patient groups.

During the vEEG recordings, postictal blood sampling following a defined seizure event (the index seizure; IS) was performed in 33 out of 56 patients (n = 16 TLE, n = 9 FLE, n = 4 TLE + PNES and n = 4 PNES (Fig. 1)). Temporal and frontal lobe IS and additional seizures before blood sampling were confirmed with adequate semiology and ictal epileptiform activity on EEG (Table 2). A majority of the temporal lobe IS consisted of focal seizures with impaired awareness (94%) with a duration of about 1.5 min. The frontal lobe IS included differential motor symptoms (tonic, myoclonic, hyperkinetic and bilateral convulsive), often nocturnally (78%), with a seizure duration of about 50 s. The amount of interictal activity (mainly graded as sparse) did not differ significantly between the TLE and FLE groups (p = 0.85). Patients with PNES exhibited a normal postcentral background activity and lack of ictal epileptiform activity on EEG during their seizure events. The seizure semiology included varying amounts of motor activity with hypermotor (25%) and akinetic behaviour (50%), as well as subjective symptoms (25%), with a median duration of 75 s. All PNES seizures occurred during wakefulness. The number of seizures occurring after the IS but before postictal blood sampling 6–8 and/or 24 h later ranged from zero to four in both TLE and FLE patients and from zero to two in the PNES group. In 23 out of 56 patients only interictal blood sampling was achieved, mainly due to logistic difficulties. However, the TLE, FLE and PNES diagnosis were still confirmed during the vEEG monitoring, except for 2 TLE patients who underwent additional verifying investigations.

**Table 2:** Temporal and frontal lobe seizures and PNES in patients during videoEEG monitoring.

	TLE (n = 13_6 h_+3_24 h_)	FLE (n = 9_6 h_)	TLE + PNES (n = 4_6 h_)	PNES (n = 4_6 h_)
**Semiology Index seizure % (n)**	Fo_A_NM = 6 (1)	Fo_A_Mtonic = 11 (1)	A = 25 (1)	A = 50 (2)
	Fo_IA_NM = 13 (2)	Fo_A_Mhyperkinetic = 22 (2)	HM = 25 (1)	HM = 25 (1)
	Fo_IA_M = 6 (1)	Fo_A_Mmyoclonic = 11 (1)	SS = 50 (2)	SS = 25 (1)
		Fo_IA_NM = 11 (1)		
	Fo_IA_Mauto = 44 (7)	Fo_IA_Mtonic = 22 (2)		
		Fo_IA_Mhyperkinetic = 11 (1)		
	Fo_IA_BC = 31 (5)	Fo_IA_BC = 11 (1)		
**Duration of index seizure: median sec (IQR)**	90 (67–143)	50 (13–68)	75 (45–131)	73 (16–298)
**Index seizure during sleep % (n)**	40 (6)	78 (7)	0 (0)	0 (0)
**Seizures between Index and postictal blood sampling: median no (range)**	6 h: 1 (0–4) incl. n = 4 Fo_IA_BC	6 h:1 (0–4)	6 h: 0 (0–1)	0.5 (0–2)
	24 h: 1.5 (0–4) incl. n = 1 Fo_IA_BC<			
**Time between last seizure and postictal blood sampling: median h (IQR)**	4.3 (2.2–6.5)	3.6 (0.6–5.9)	6.1 (6.0–6.2)	1.0 (0.8–5.5)
**Interictal activity grading 0**–**3: median (IQR)**	1 (0–2)	1 (0–3)	0 (0–1.5)	0 (0–0)

### 2.2 Interictal increase in IL-6 in serum in patients with TLE and FLE but not PNES

**Fig. 2:**
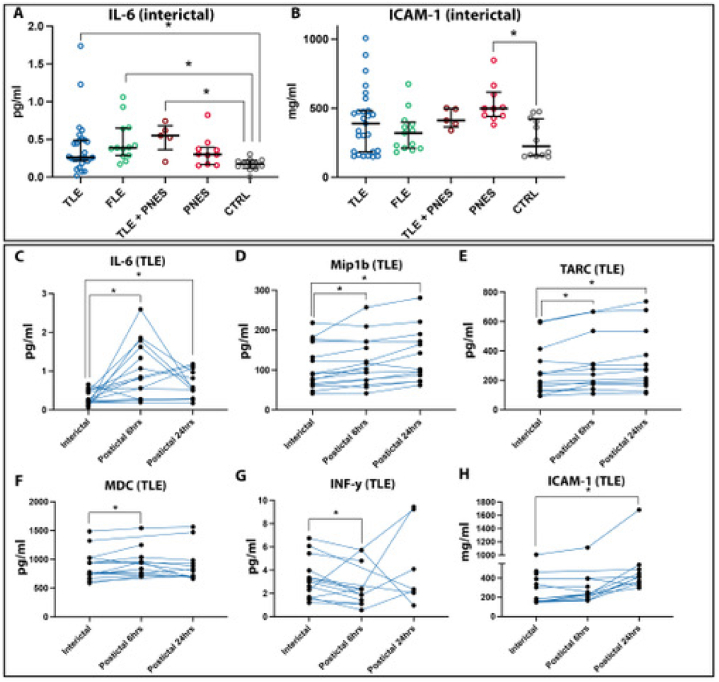
**Interictal and postictal immune protein levels in serum from patients with temporal and frontal lobe seizures and PNES.** A-B, interictal IL-6 and ICAM-1 protein levels in patients with TLE, FLE, TLE + PNES, PNES, and controls (CTRL). C–H, interictal compared to postictal protein levels of IL-6, MIP1β, TARC, MDC, IFN-y, ICAM-1at 6–8 h and 24 h after temporal lobe seizures. Kruskal Wallis H-test was used in A and B and Wilcoxon paired test in C–H. p-values ≤0.05 were considered statistically significant.

On admission day for vEEG recordings, interictal levels of IL-6 were increased in the TLE, FLE and TLE + PNES group compared to controls (p = 0.038, p = 0.002 and p = 0.009, respectively, Table 3, Fig. 2A). This increase was absent in the PNES group. In contrast, the PNES group exhibited increased interictal Intercellular Adhesion Molecule-1(ICAM-1) levels compared to controls (p = 0.006, Fig. 2B), while no changes were observed in the TLE or FLE group. The PNES patients had higher levels of interictal Macrophage-derived Chemokine (MDC) levels compared to the TLE group (p = 0.01), though, none of them were significantly different compared to the control group. Interictal levels of **the affected immune factors** IL-6 and ICAM-1 did not correlate to self-reported seizure frequency and severity during the last six months, time since last seizure, presence of comorbidities, MRI findings, or numbers of current ASMs, except for ICAM-1 levels and **self-reported** seizure frequency in FLE patients (Supplementary T. 1). **Correlations were also made for age, gender, sleep disturbances and physical activity, without showing**

**Table 3:** Interictal immune protein levels in serum from patients with TLE, FLE and PNES.

Variable	TLE (n)	FLE (n)	TLE with PNES (n)	PNES (n)	Control (n)	P-value
**CRP (mg/ml)**	570, 170–1370 (27)	1120, 300–2310 (13)	690, 420–2920 (5)	520, 380–1460 (10)	160, 90–1160 (12)	0.32
**Eotaxin (pg/ml)**	220, 170–320 (28)	210, 150–240 (13)	240, 150–310 (5)	210, 140–230 (10)	240, 200–270 (12)	0.64
**Eotaxin-3 (pg/ml)**	7.5, 5.8–11 (26)	9.8, 6.2–14 (12)	16, 3.9–96 (5)	10, 8.8–13 (8)	9.3, 7.2–13 (12)	0.44
**Fraktalkine (ng/ml)**	0.6, 0.5–0.8 (12)	*N.D*	*N.D*	0.51, 0.49–0.60 (5)	0.56, 0.50–0.62 (6)	0.45
**ICAM (mg/ml)**	390, 180–480 (27)	320, 210–400 (13)	410, 360–500 (5)	500, 440–620 (10)	220, 160–420 (12)	**0.01**
**IL-1b (pg/ml)**	*N.D*	*N.D*	*N.D*	*N.D*	*N.D*	–
**IL-2 (pg/ml)**	0.09, 0.06–0.3 (12)	0.06, 0.02–0.08 (4)	*N.D*	0.06, 0.11, 0.15 (3)	0.09, 0.04–0.20 (6)	0.38
**IL-4 (pg/ml)**	0.02, 0.02–0.03 (8)	0.02, 0.007–0.03 (6)	0.003, 0.01, 0.01 (3)	0.02, 0.01–0.05 (4)	0.009, 0.004–0.01 (5)	0.052
**IL-6 (pg/ml)**	0.26, 0.22–0.49 (28)	0.39, 0.29–0.64 (13)	0.55, 0.36–0.68 (5)	0.30, 0.17–0.39 (10)	0.17, 0.12–0.22 (12)	**0.001**
**IL-8 (pg/ml)**	7.2, 5.1–9.4 (28)	5.8, 4.2–7.8 (13)	5.3, 4.3–6.8 (5)	6.6, 4.0–8.9	5.4, 4.9–6.6 (12)	0.37
**IL-10 (pg/ml)**	0.22, 0.16–0.40 (28)	0.23, 0.15–0.51 (13)	0.49, 0.10–0.72 (5)	0.14, 0.13–0.21 (7)	0.19, 0.13–0.25 (12)	0.14
**IL-12p70 (pg/ml)**	0.09, 0.05–0.19 (18)	0.20, 0.04–0.56 (8)	*N.D*	0.09, 0.61–1.8 (5)	0.51, 0.05–0.72 (5)	0.64
**IL-13 (pg/ml)**	0.78, 0.41–1.4 (13)	0.62, 0.48–1.4 (5)	*N.D*	*N.D*	1.5, 0.67–1.6 (5)	0.50
**INF-y (pg/ml)**	2.6, 1.3–3.3 (19)	3.5, 2.3–17 (12)	2.8, 2.1–3.9 (4)	3.04, 1.17–9.07 (7)	2.9, 2.0–3.4 (11)	0.46
**IP-10 (pg/ml)**	150, 110–180 (28)	120, 80–220 (13)	180, 80–240 (5)	190, 120–220 (10)	160, 90–170 (12)	0.68
**KC/Gro (pg/ml)**	80, 60–90 (25)	*N.D*	*N.D*	80, 70–100 (10)	80, 60–120 (12)	0.83
**MCP-1 (pg/ml)**	200, 160–260 (28)	190, 160–250 (13)	230, 150–260 (5)	190, 130–280 (10)	180, 130–320 (12)	0.97
**MCP-4 (pg/ml)**	90, 60–110 (28)	80, 70–120 (13)	90, 80–120 (5)	90, 70–100 (10)	80, 60–120 (12)	0.94
**MDC (pg/ml)**	770, 700–1010 (28)	1040, 650–1350 (13)	1300, 700–1330 (5)	1270, 1000–1440 (10)	1030, 930–1120 (12)	**0.014**
**Mip1a (pg/ml)**	16, 12–19 (24)	15, 9.2–19 (10)	14, 9.6–18 (5)	13, 10–14 (7)	14, 12–22 (11)	0.57
**Mip1b (pg/ml)**	90, 60–140 (28)	100, 60–120 (13)	90, 70–110 (5)	90, 60–110 (10)	80, 60–110 (12)	0.74
**SAA (mg/ml)**	810, 400–2180 (27)	720, 400–1250 (13)	530, 300–4410 (5)	640, 410–1550 (10)	600, 290–3070 (12)	0.99
**TARC (pg/ml)**	240, 140–320 (28)	210, 150–540 (13)	270, 190–290 (5)	310, 190–470 (10)	290, 160–400 (12)	0.63
**TNFa (pg/ml)**	2.0, 1.8–2.5 (27)	2.4, 1.9–2.7 (12)	2.3, 1.9–2.8 (5)	1.9, 1.4–2.7 (5)	2.3, 1.8–2.6 (12)	0.64
**VCAM (mg/ml)**	510, 460–850 (27)	550, 440–710 (13)	600, 390–780 (5)	530, 480–710 (10)	590, 550–640 (12)	0.81

**any correlation to interictal IL-6 or ICAM-1 levels.** The additional 22 immune factors showed no difference between patient and control groups (Table 3). However, a few protein levels, IL-1β in particular, were not determined due to levels below detection limits and keratinocyte chemo attractant/human growth-regulated oncogene (KC/GRO) and fractalkine, were only analyzed in the TLE, PNES and control group.

### 2.3 Postictal immune reaction in serum after temporal but not frontal lobe seizures or PNES

At 6–8 h after an index seizure, TLE patients exhibited increased postictal levels of IL-6, MDC, Macrophage Inflammatory Protein 1B (MIP1β) and Thymus- and Activation-regulated Chemokine**(**TARC) and a decrease in Interferon-y (INF-y) compared to the interictal levels (Table 4 and Fig. 2C-G). IL-6, MIP1β and TARC remained elevated at 24 h (Fig. 2C-E). ICAM-1 levels were also increased but only at 24 h (Fig. 2H). The postictal alterations of IL-6, MDC, TARC, INFy and ICAM-1 in TLE patients did not correlate to the duration or severity of seizures, or time between additional seizures and blood sampling, or to the amount of interictal activity. MIP1β levels correlated to the number of focal evolving into bilateral convulsive seizures occurring between an index temporal lobe seizure and blood sampling at 6–8 h. However, only 4 TLE patients exhibited these additional generalized seizures, which makes the correlation analyses uncertain. (Supplementary T. 2). No changes in postictal immune factors compared to interictal levels were detected in FLE patients (Table 4). In the TLE + PNES and PNES patients, only a limited number of postictal samples were collected. Where statistics could be performed the p-values were >0.05 (Table 4).

**Table 4:** Postictal immune protein levels in serum from patients with temporal and frontal lobe seizures and PNES.

	TLE (n)	FLE (n)	TLE with PNES (n)	PNES (n)
**Ratio of postictal protein levels 6 h after index seizure to interictal levels**				
**CRP**	1.1, 0.9–2.0 (13) p = 0.15	1.1, 0.80–1.9 (9) p = 0.43	0.9, 0.7–1.1 (4) p = 0.38	0.7, 0.5–0.8 (4) p = 0.13
**Eotaxin**	0.9, 0.8–1.1 (13) p = 0.13	1.0, 0.8–1.1 (9) p = 0.91	1.0, 0.9–1.1 (4) p > 0.99	0.9, 0.9–1.1 (4) p = 0.63
**Eotaxin-3**	1.0, 0.9–1.1 (13) p = 0.74	1.1, 1.0–1.3 (9) p = 0.30	1.0, 0.7–1.3 (4) p > 0.99	0.9, 0.9–1.1 (4) p = 0.63
**Fraktalkine**	1.2, 0.9–1.4 (12) p = 0.23	*N.D*	*N.D*	0.9, 0.7–1.3 (4) p = 0.88
**ICAM-1**	1.1, 1.0–1.3 (13) p = 0.13	1.1, 1.0–1.2 (9) p = 0.13	0.9, 0.8–1.0 (4) p = 0.38	0.9, 0.7–1.1 (4) p = 0.38
**IL-1b**	*N.D*	*N.D*	*N.D*	*N.D*
**IL-2**	*N.D*	*N.D*	*N.D*	*N.D*
**IL-4**	*N.D*	*N.D*	*N.D*	*N.D*
**IL-6**	2.7, 1.2–11 (13) **p** = **0.003**	0.9, 0.5–2.3 (8) p = 0.65	1.1, 1.0–1.5 (4) p > 0.99	0.9, 0.9–1.1 (4) p = 0.38
**IL-8**	1.2, 0.00–1.4 (13) p = 0.27	1.1, 0.8–1.5 (9) p = 0.50	0.9, 0.8–1.1 (4) p = 0.75	1.1, 0.9–1.1 (4) p = 0.38
**IL-10**	1.1, 1.0–1.5 (13) p = 0.27	0.8, 0.7–1.3 (9) p = 0.25	*N.D*	*N.D*
**IL-12p70**	*N.D*	*N.D*	*N.D*	*N.D*
**IL-13**	*N.D*	*N.D*	*N.D*	*N.D*
**INF-y**	0.7, 0.6–0.9 (11) **p** = **0.007**	1.0, 0.4–1.6 (8) p = 0.64	*N.D*	*N.D*
**IP-10**	1.0, 0.8–1.2 (13) p = 0.68	1.2, 1.0–1.7 (9) p = 0.16	1.3, 0.8–1.8 (4) p > 0.99	1.1, 0.8–1.4 (4) p = 0.63
**KC/Gro**	1.0, 0.9–1.1 (12) p = 0.81	*N.D*	*N.D*	1.1, 1.0–1.1 (4) p = 0.25
**MCP-1**	1.0, 0.7–1.2 (13) p = 0.74	1.0, 0.9–1.2 (9) p > 0.99	1.0, 0.8–1.1 (4) p = 0.63	1.0, 0.9–1.1 (4) p > 0.99
**MCP-4**	1.0, 0.9–1.3 (12) p = 0.50	1.1, 0.9–1.4 (9) p = 0.25	1.1, 0.9–1.4 (4) p = 0.63	0.9, 1.1–1.2 (4) p = 0.63
**MDC**	1.1, 1.0–1.2 (13) **p** = **0.006**	1.1, 1.0–1.1 (9) p = 0.25	1.0, 1.0–1.1 (4) p > 0.99	0.9, 0.9–1.2 (4) p = 0.88
**Mip1a**	1.0, 0.9–1.2 (12) p = 0.79	1.1, 0.9–1.4 (7) p = 0.94	*N.D*	*N.D*
**Mip1b**	1.1, 1.0–1.3 (13) **p** = **0.04**	1.1, 0.9–1.2 (9) p = 0.43	1.0, 1.0–1.1 (4) p = 0.88	1.0, 1.0–1.2 (4) p = 0.88
**SAA**	1.2, 0.9–1.7 (13) p = 0.27	1.5, 0.9–2.2 (9) p = 0.16	1.0, 0.7–1.1 (4) p = 0.88	0.9, 0.5–1.1 (4) p = 0.36
**TARC**	1.1, 1.0–1,2 (13) **p** = **0.005**	1.1, 1.0–1.2 (9) p = 0.05	1.0, 1.0–1.1 (4) p = 0.63	0.9, 0.9–1.2 (4) p > 0.99
**TNFa**	1.0, 0.9–1.1 (12) p = 0.84	1.1, 1.0–1.2 (9) p = 0.30	0, 0.9–1.1 (4) p = 0.50	1.1, 1.0–1.1 (4) p = 0.38
**VCAM**	1.2, 1.0–1.3 (13) p = 0.17	1.0, 1.0–1.1 (9) p = 0.36	0.9, 0.7–1.1 (4) p = 0.63	0.9, 0.7–1.1 (4) p = 0.25
**Ratio of postictal protein levels 24 hrs after index seizure to interictal levels**				
**CRP**	0.6, 0.2–3.8 (13) p > 0.99	0.8, 0.2–4.4 (6) p = 0.69	*N.D*	0.4, 0.7, 0.8 (3) p = 0.25
**Eotaxin**	0.9, 0.7–1.2 (13) p = 0.27	0.8, 0.6–1.0 (6) p = 0.22	*N.D*	0.8, 0.9, 1.2 (3) p = 0.50
**Eotaxin-3**	1.0, 0.6–1.1 (11) p = 0.21	1.1, 0.9–1.9 (6) p = 0.56	*N.D*	1.2, 1.2, 1.2 (3) p = 0.25
**Fraktalkine**	*N.D*	*N.D*	*N.D*	*N.D*
**ICAM-1**	2.0, 1.2–2.5 (13) **p** = **0.005**	2.0, 1.2–2.3 (7) p = 0.16	*N.D*	0.9, 1.1, 1.2 (3) p > 0.99
**IL-1b**	*N.D*	*N.D*	*N.D*	*N.D*
**IL-2**	*N.D*	*N.D*	*N.D*	*N.D*
**IL-4**	*N.D*	*N.D*	*N.D*	*N.D*
**IL-6**	1.9, 1.2–4.0 (12) **p** = **0.002**	1.1, 0.7–2.0 (5) p = 0.44	*N.D*	0.6, 1.0, 2.4 (3) p > 0.99
**IL-8**	0.9, 0.9–1.2 (12) p = 0.42	0.8, 0.6–1.0 (6) p = 0.09	*N.D*	1.2, 1.4, 1.5 (3) p = 0.25
**IL-10**	1.0, 0.6–1.2 (10) p = 0.43	1.0, 0.7–1.6 (5) p > 0.99	*N.D*	*N.D*
**IL-12p70**	*N.D*	*N.D*	*N.D*	*N.D*
**IL-13**	*N.D*	*N.D*	*N.D*	*N.D*
**INF-y**	1.0, 0.4–2.8 (7) p = 0.81	0.4, 0.4, 0.5 (3) p = 0.25	*N.D*	*N.D*
**IP-10**	1.0, 0.9–1.1 (13) p = 0.95	1.1, 0.9–1.9 (7) p = 0.21	*N.D*	1.1, 1.1, 1.5 (3) p = 0.25
**KC/Gro**	*N.D*	*N.D*	*N.D*	*N.D*
**MCP-1**	1.0, 0.9–1.2 (13) p = 0.68	0.9, 0.7–1.0 (6) p = 0.22	*N.D*	0.9, 1.1, 1.5 (3) p = 0.75
**MCP-4**	0.9, 0.8–1.0 (13) p = 0.22	0.9, 0.7–1.9 (6) p = 0.84	*N.D*	0.9, 1.2, 1.2 (3) p = 0.50
**MDC**	1.0, 0.9–1.3 (13) p = 0.79	0.9, 0.9–1.2 (7) p = 0.47	*N.D*	0.9, 1.1, 1.2 (3) p = 0.75
**Mip1a**	1.3, 0.8–1.6 (8) p = 0.74	0.9, 0.9–1.3 (5) p > 0.99	*N.D*	*N.D*
**Mip1b**	1.3, 1.0–1.5 (13) **p** = **0.0005**	1.2, 0.8–1.5 (6) p = 0.56	*N.D*	1.1, 1.1, 1.1 (3) p = 0.25
**SAA**	0.7, 0.3–5.5 (13) p = 0.95	0.6, 0.4–4.8 (7) p = 0.81	*N.D*	0.7, 0.9, 1.1 (3) p = 0.75
**TARC**	1.1, 1.1–1.2 (13) **p** = **0.0007**	1.0, 0.9–1.3 (6) p = 0.56	*N.D*	0.9, 0.9, 1.0 (3) p > 0.99
**TNFa**	1.0, 0.9–1.2 (12) p = 0.62	1.0, 1.0–1.2 (6) p = 0.69	*N.D*	1.2, 1.2, 1.4 (3) p = 0.25
**VCAM**	0.9, 0.8–1.1 (13) p = 0.64	0.8, 0.8–1.1 (7) p = 0.16	*N.D*	0.9, 0.9, 1.1 (3) p = 0.50

## 3 Discussion

In the present study we describe increased interictal serum levels of IL-6 in both TLE and FLE patients compared to control subjects. This is in contrast to patients with vEEG-verified PNES who showed no increase in IL-6, but increased interictal ICAM-1 levels.

In addition, we present a new combination of six immune factors in blood that react within 6–24 h after a vEEG-verified temporal lobe seizure in adult patients. It is comprised of an increased postictal/interictal ratio of IL-6, MIP1β, TARC, MDC and ICAM-1 and decreased IFN-y levels. These fluctuations in serum levels between interictal and postictal period were not observed in patients with frontal lobe seizures or in single cases with PNES.

Although our study showed alterations of IL-6, MIP1β, TARC, MDC, ICAM-1 and IFN-y in peripheral blood, it can still be hypothesized that the immune markers have entered the blood stream from the central nervous system [20]. Innate immune reactions affect the integrity of the blood-brain barrier (BBB), which allows peripheral immune cells and albumin to cross the BBB and the oxidative stress induced by epileptic seizures may also affect the activation of circulating white blood cells [20,21]. A neurovascular unit dysfunction and the resulting inflammatory process is believed to have an important role in late-onset epilepsy [22]. Future studies assessing immune factors and white blood cell activation by FACS are needed to further describe the interaction between the central nervous system and the peripheral immune system.

We are, to our knowledge, the first to report elevated interictal serum levels of IL-6 in patients with vEEG-verified FLE. Data from FLE patients have been included in previous studies but merged with other focal seizures in the heterogenous group of XTLE patients for which both increased and unchanged IL-6 levels have been reported [13,17]. Previous studies of IL-6 in TLE patients have also been diverse, but include reports of elevated interictal IL-6 levels [[14], [15], [16],^23^]. Increased interictal IL-6 levels in TLE and FLE were in our study not confounded or correlated to either patient-reported seizure burden, comorbidities, 1.5–3T MRI brain findings, or current number of ASMs. This is in line with two previous studies on TLE patients in hospital settings where IL-6 levels could also not be correlated to disease duration, seizure frequency, or ASM class [15,24]. However, in a large retrospective study of 1218 patients with epilepsy, interictal IL-6 levels were positively correlated to seizure frequency and severity in both TLE, XTLE and idiopathic generalized epilepsy [11]. In addition to the fact that the fewer number of patients included in our study makes strong correlations analyses more difficult to achieve, this discrepancy may be due to selection bias. We included patients undergoing vEEG investigation for medically refractory seizures, higher seizure burden or diagnostic challenges, while Wang et al. also included non-refractory patients [11]. Consequently, patients with effective seizure treatment were left out in our study. A comparison of interictal IL-6 levels in patients with medically refractory epilepsy to those with appropriate seizure control is yet to be performed. **Decreased levels of IL-6 and IL-1b have been found after surgical treatment of drug-resistant TLE** [25]**, indicating that epileptogenesis may in itself induce/maintain inflammatory activity. Inflammatory processes have also been shown to have a role in epileptogenesis, and studies on anti-inflammatory agents targeting specific inflammatory pathways have offered promising results in the treatment of drug-resistant epilepsy** [2,26,27].

In spite of the relatively high percentage of comorbidities within the present patient cohorts (15–60%) and previous publications suggesting increased IL-6 levels in i.e. depression, insomnia and sleep apnea, which are frequent comorbidities in epilepsy [[28], [29], [30], [31]], no neurological/psychiatric/neuropsychiatric co-morbidities correlated to the increased interictal IL-6 levels in the TLE and FLE patients. IL-6 serum levels are also known to fluctuate due to physical activity and muscle contractions [32]. However, this is less likely a significant confounder in the present study as self-reported amount of physical activity was generally low and few patients exercised during the vEEG monitoring.

Previous publications have defined the postictal time period as <24–48 h post-seizure and the interictal period as >24 h–7 days post-self-reported seizure [11,12]. However, these arbitrary definitions are difficult to verify without continuous vEEG but of importance when interpreting and detecting transient seizure-induced immune responses. As an example, Alapirtti et al., 2018 noted that lower levels of interictal IL-6, as well as a lower seizure frequency in TLE patients during the year prior to the vEEG monitoring, correlated to a significantly higher increase in postictal IL-6 [15]. It could suggest that higher interictal levels rather than lower postictal levels explains the absence of IL-6 increase during frequent epileptic seizures, **as in the case of the FLE group in our study. The FLE patients showed no postictal increase of IL-6, and their self-reported seizure frequency of 5.5 per week was considerably higher than in the TLE group (0.75 per week). T**he majority of FLE patients exhibited daily nocturnal seizures during their continuous vEEG recordings, which may have resulted in frequent re-occurring postictal periods and few interictal periods which then explains the lack of increase in IL-6 postictal/presumed interictal ratio, **possibly resulting in chronically elevated IL-6 levels. This proposed mechanism should be verified in future studies by assessing leukocyte activation, as it has been shown to fluctuate in relation to epileptic seizures** [33]. Altogether, the results emphasize the need for vEEG verified ictal/postictal/interictal periods.

The increased ratio of postictal/interictal levels of IL-6 6–8 h post-temporal lobe seizures could not be correlated to the duration or severity of the IS or additional seizures occurring before blood sampling. This is in line with a few previous studies, but in contrast to a vEEG study where patients showed higher postictal IL-6 levels after more severe seizures with bilateral tonic-clonic movements and if the IS lasted longer than 100 s [15,16,24]. In addition to seizure parameters, interictal epileptiform activity on EEG did also not correlate to the postictal/interictal IL-6 levels. However, it is possible that the amount of interictal activity evaluated during the first 3 days of the vEEG monitoring was confounded by various degree of ASM withdrawal at hospital admission, performed in order to provoke and diagnose seizures. More accurate correlation analyses between IL-6 levels and estimated amount of interictal epileptiform activity could in future studies be performed during continued ASM treatment. It is possible that the ASM withdrawal also introduced a somewhat higher diversity in the immune response among patients, as ASMs such as carbamazepine are known to alter some cytokine levels [34]. This could have concealed minor changes in additional immune factors apart from the now described changes in TARC, MDC, MIP1β, or INF-y levels. A trend for increased TARC levels and TARC/sICAM5 ratio after focal seizures has previously been suggested, but also an increase in INF-y after temporal lobe seizures, the latter finding being in contrast to the decreased postictal/interictal ratio of INF-y levels observed in our study [12,35,36].

PNES occur with a prevalence of about 12% in people with epilepsy [37]. **An increase in ICAM-1 expression after TLE seizures has been reported in epilepsy research and is associated with endothelial activation and leukocyte recruitment across the BBB** [[38], [39], [40]]. A recent study suggests evaluation of ICAM-1 and TRAIL levels in **postictal** serum when differentiating epileptic seizures and PNES [41]. **Our data suggests that this difference is due to an interictal elevated level of ICAM-1 in PNES patients. P**revious studies have also reported elevated levels of ICAM-1 in CSF or serum in psychiatric disorders such as schizophrenia and major depressive disorder [42,43]. Although ICAM-1 levels may be affected by medication, in particular antipsychotics, ICAM-1 is mainly perceived as a marker of a blood brain barrier disruption and an inflammatory process in psychiatric disorders [44]. We found increased interictal levels of ICAM-1 in PNES patients without confirmed epilepsy, but cannot exclude that psychiatric comorbidities may have confounded this finding. **The deviating ICAM-1 measures may indicate that dysregulation of the permeability of the BBB is a shared trait in numerous psychiatric disorders** [44]. The accurate diagnosis of PNES by a biomarker with acceptable specificity and sensitivity remains a challenge. The lack of an increase in IL-6 following PNES is the most robust observation in the current study.

Our findings support the notion that immune factors in blood have the potential to be future biomarkers for epileptic seizures and a relevant tool for the diagnosis of various clinical presentations associated with different epileptic seizures and PNES. The heterogeneity among different epileptic seizures may be disclosed in peripheral blood sampling independent of co-morbidities.

## 4 Materials and methods

### 4.1 Study design and research project participants

Patients admitted to the epilepsy monitoring unit in Skane or Uppsala University Hospital for diagnostic purposes were included in the study (Fig. 1). Inclusion criteria were age >18 and suspected or confirmed diagnosis of epileptic and/or PNES. Exclusion criteria were age <18, intracranial EEG monitoring, head trauma or surgery within the last six months, presence of symptomatic systemic immune disease and regular intake of immune modulating medication. **All patients were considered to have drug-resistant seizures, e.g. trial of 2 or more ASMs at appropriate dosages**. Medical records provided information on MRI findings and medical history. At inclusion, a questionnaire was administered to collect self-reported information on seizure semiology, exercise habits and co-morbid disorders. Physical activity was defined as 30 min episode of physical activity with heart rate above 100 beats/min (equivalent to a quick walk) and graded 1–3 with grade 1 = never, grade 2 = 1–3 times/week, grade 3= >3 times/week.

The patients underwent three to five days of continuous vEEG monitoring with 24 h medical supervision. Seizure semiology was classified by operational criteria in accordance with the International League Against Epilepsy (ILAE) [45]. The EEG adhered to the international standard 10–20 electrode placement, consisted of 32 or 64 electrodes, and was recorded as a bipolar montage. Interictal activity was graded as 0 = no interictal activity, 1 = sparse interictal activity with a few spikes/sharp waves observed during a 20 min period, 2 = moderate interictal activity including everything between grade 1 and 3, and 3 = abundant interictal activity with spikes/sharp waves observed every minute. Based on the results from vEEG recordings, patients were assigned to the following groups: TLE, FLE, TLE + PNES or only PNES. Previous EEG recordings and medical records were used to support or exclude epilepsy diagnosis in patients that had exclusively PNES during the vEEG monitoring, **thus taking into account the complexity of diagnosing PNES and acknowledging the risk of comorbidity in epilepsy patients** [46,47].

Age and gender matched, healthy individuals served as controls and submitted the same medical history questionnaire.

The study design was approved by the Malmö-Lund and Uppsala ethical committees in Sweden (number 2016/85 and 2016–85, respectively) and in line with the Helsinki declaration. Written informed consent was obtained from all participants.

### 4.2 Collection of blood samples

Blood samples from patients were collected at three occasions. A baseline blood sample (interictal) was taken at the time of enrolment in the study, shortly (about 6 h) after admission to the epilepsy monitoring unit in patients negating seizures within the last 24 h. Swift verification of seizures based on the results from the ongoing vEEG allowed for renewed blood sampling at 6–8 and 24 h after an IS (postictally). Control subjects provided only baseline samples. After sample collection in a 5 ml BD Vacutainer serum tube, blood samples were incubated at room temperature for 1 h before centrifugation at 2500 rpm for 15 min. Separated serum samples were aliquoted and stored at −80 °C until analysis.

### 4.3 Biochemical analyses

Serum concentrations of 23 cytokines and chemokines were determined via Multiplex ELISA proinflammatory panel 1 (INF-γ, IL1-β, IL-2, IL-4, IL-6, IL-8, IL-10, IL-12p70, IL-13 and TNF-α), chemokine panel 1 (Eotaxin, Eotaxin-3, MIP-1α, MIP-1β, TARC, IP-10, MCP-1, MCP-4 and MDC) and vascular injury panel 2 (SAA, CRP, VCAM-1 and ICAM-1) human kits from Mesoscale according to the manufacturer instructions (Mesoscale, US). Briefly, serum samples were diluted 1:2, 1:4 or 1:1000 and loaded in duplicates into the MSD plate together with the assigned standard. Plates were either incubated on a shaker with serum samples at 4 °C overnight, or for 2 h in room temperature. They were then washed, and detection antibody was added for 2 h at room temperature, followed by washing again before adding read buffer. Plates were read using the MSD MESO QuickPlex SQ and the protein concentration was calculated. All samples with a CV% constant above 40 were excluded from all comparisons.

Serum concentration of two additional chemokines, KC/Gro and fractalkine, were determined with ELISA human Quantikine plates according to the manufacturer's instruction (RD Systems, US). Briefly, undiluted serum was loaded together with assay diluent as singles and incubated for 2 h at room temperature. Plates were washed and incubated with secondary antibodies for 1–2 h at 4 °C. After the second wash substrate solution was added and incubated for 30 min. Reading of absorbance occurred instantly after stop solution was added using the ASYS Expert 96 plate reader at 450 nm.

### 4.4 Statistical analyses

ELISA data is expressed as mean with standard deviation, or median with interquartile range. All data were tested for normality using the Shapiro-Wilk test. Detailed statistical data is presented in Supplementary T. 1. Upon non-normal distribution of data, 2 group-comparisons were performed with Mann-Whitney *U* test and ≥3 group-comparisons with Kruskal Wallis H-test. For normally distributed data unpaired Student's t-test was used for 2 group-comparisons and one-way ANOVA with Bonferroni post hoc test for ≥3 group-comparisons. Binary outcomes (gender, reported comorbidity, MRI findings, reported sleep disturbance, occurrence of seizures during night/sleep) were compared with Fisher exact test. Correlation analysis of continuous data was evaluated with non-parametric Spearman correlation. Post-ictal protein levels were compared to interictal levels from the same patient with Wilcoxon paired test. All p-values <0.05 were considered statistically significant.

## Author contributions

Matilda Ahl and Marie K Taylor: Conceived and designed the experiments; Performed the experiments; Analyzed and interpreted the data; Contributed reagents, materials, analysis tools or data; Wrote the paper.

Una Avdic: Conceived and designed the experiments; Performed the experiments; Wrote the paper. Anna Lundin: Analyzed and interpreted the data; Wrote the paper.

My Andersson: Contributed reagents, materials, analysis tools or data; Wrote the paper.

Åsa Amandusson and Eva Kumlien: Contributed reagents, materials, analysis tools or data; Wrote the paper.

Maria Compagno Strandberg: Conceived and designed the experiments; Analyzed and interpreted the data; Contributed reagents, materials, analysis tools or data; Wrote the paper.

Christine T Ekdahl: Conceived and designed the experiments; Performed the experiments; Analyzed and interpreted the data; Contributed reagents, materials, analysis tools or data; Wrote the paper.

## Funding statement

This work was supported by 10.13039/501100004359Swedish Research Council (523-2012-2297), Swedish ALF Grant for funding for medical training and research (RSID 130567), The Crafoord Foundation (20140857), and the Elsa Schmitz foundation (2020/664).

## Data availability

All study data is available from the corresponding author upon reasonable request.

## Declaration of competing interest

The authors declare no competing interests.

## Appendix A Supplementary data

Questionnaire_Research project participants Questionnaire_Research project participants

Supplementary Material Supplementary Material

Questionnaire_Research project participants_English Questionnaire_Research project participants_English
