# Supplementary material for: Immune response in blood before and after epileptic and psychogenic non-epileptic seizures
Source: Heliyon. 2023 Feb 21;9(3):e13938. doi: 10.1016/j.heliyon.2023.e13938 (PMC9988551; doi:10.1016/j.heliyon.2023.e13938)
Supplement: Questionnaire_Research project participants [file mmc1.pdf]

Frågeformulär /Questionnaire

English translation below

Frågeformulär till försöksperson vid inskrivning för video-EEG-monitorering

1. När hade du anfall senast?

2. Hur många anfall per vecka har du haft senaste halvåret respektive senaste månaden?

3.Vilka symtom har du under dina anfall? Kan du beskriva hur ett av dina typiska anfall känns?

4. Hur mycket tränar du i veckan (så att pulsen går över 100slag/min i >30 min)? Ringa in ett svar.

Aldrig 1-3ggr/vecka >3ggr/vecka

5. Hur mycket alkohol har du druckit senaste månaden? Ringa in ett svar.

Alternativ 1. <1 glas vin eller 1 starköl/vecka Alternativ 2. Mellan alternativ 1 och 3

Alternativ 3. Kvinnor: >2 flaskor vin eller 10 flaskor starköl/v Män: >3 flaskor vin eller 15 flaskor starköl/v

6. Har du slagit i huvudet så att du svimmat senaste 6 månaderna?

7. Har du genomgått en hjärnoperation? Om JA – när gjorde du det?

8. Har du en pågående inflammatorisk sjukdom, tex inflammation i nerver eller leder – vilken?

9. Har du en pågående neurologisk eller psykiatrisk sjukdom – vilken?

____

10. Finns det någon ärftlig sjukdom i din släkt? Har du någon ärftlig sjukdom – vilken?

____

11. Har du en diagnostiserad sömnstörning – vilken?

____

12. Har du en neuropsykiatrisk funktionsnedsättning, tex autism – vilken?

____

13. Har du genomgått elektrokonvulsiv behandling (ECT) senaste 6 månaderna?
____

14. Är du gravid?
____

15. Vilka mediciner har du tagit regelbundet senaste månaden?
____

Datum:___________________ Ort: _____________________

Questionnaire for research project participant at admittance to video-EEG monitoring

1. When was your last seizure?

   ____

2. How many seizures per week have you experienced in the last six months, and the last month, respectively?

   ____

3. Which symptoms do you experience during your seizures? Could you describe what one of your typical seizures feels like, to you?

   ____

4. How much physical exercise do you do per week (pulse above 100 bpm for a duration of >30 min)? Circle your answer:

   None 1-3 times/week >3 times/week

5. How much alcohol did you consume in the last month? Circle your answer:

Alternative 1: < 1 glass of wine or 1 strong beer/week

Alternative 2: In between alternatives 1 and 3.

Alternative 3: Women: > 2 bottles of wine or 10 bottles of strong beer/week, Men: >3 bottles of wine or 15 bottles of strong beer/week

6. Have you hit your head so badly you fainted, within the last six months?

7. Have you undergone brain surgery? If YES, when?

8. Do you have a current inflammatory disease, such as inflammation in nerves or joints? If so, which one?

9. Do you have a current neurological or psychiatric disorder? If so, which one?

10. Do you have any hereditary disorders in your family? Do you have a hereditary disorder?

11. Do you have a diagnosed sleep disorder?

12. Do you have a diagnosed neuropsychiatric disorder, e. g. autism?

13. Have you received ECT(Electroconvulsive Therapy) within the last six months?

14. Are you pregnant?

____

15. Which medications have you been taking regularly in the last month?

____

Date:____________________ Location:________________________
