# Supplementary material for: Immune response in blood before and after epileptic and psychogenic non-epileptic seizures
Source: Heliyon. 2023 Feb 21;9(3):e13938. doi: 10.1016/j.heliyon.2023.e13938 (PMC9988551; doi:10.1016/j.heliyon.2023.e13938)
Supplement: Supplementary Material [file mmc2.pdf]

Supplementary Material

Supplementary Table 1: Correlation analyses between interictal IL-6 and ICAM-1 levels and self-reported and medical parameters on admission to videoEEG.

Data presented as p-value with the number of patients included in each analysis (n). [1]= Spearman correlation, [2]= Mann-Whitney U-test. p-values ≤0.05 were considered statistically significant. No = number.

	TLE (n)	FLE (n)	TLE with PNES (n)	PNES (n)
**Correlation analysis to interictal IL-6 levels**				
**Reported seizure frequency last 6 months (no/week)**	p=0.98[1] (28)	p=0.45[1] (13)	p=0.81[1] (5)	p=0.30[1] (9)
**Reported last seizure (days)**	p=0.67[1] (28)	p=0.64[1] (13)	p=1.0[1] (5)	p=0.49[1] (10)
**Reported bilateral convulsive seizure last 6 months (yes/no)**	p=0.50[2] (28)	-	p=1.0[2] (3)	p=0.11[2] (9)
**Comorbidities (yes/no)**	p=0.85[2] (28)	p=0.31[2] (13)	p=0.80[2] (5)	p=0.26[2] (10)
**MR-finding (yes/no)**	p=0.73[2] (28)	p=0.46[2] (13)	p=0.20[2] (5)	p=0.45[2] (10)
**AEDs (No)**	p=0.50[1] (28)	p=0.53[1] (13)	p=0.80[1] (5)	p=0.47[1] (10)
**Correlation analysis to interictal ICAM-1 levels**				
**Reported seizure frequency last 6 months (nr/week)**	p=0.29[1] (27)	**p=0.017**[1] (13)	p=0.09[1] (5)	p=0.97[1] (10)
**Reported last seizure (days)**	p=0.11[1] (27)	p=0.07[1] (13)	p=0.42[1] (5)	p=0.63[1] (10)
**Reported bilateral convulsion (yes/no)**	p=0.88[2] (27)	-	p=0.67[2] (3)	p=1.0[2] (9)
**Comorbidities**	p=1.0[2] (27)	p=0.51[2] (13)	p=0.40[2] (5)	p=0.07[2] (10)
**MR-finding (yes/no)**	p=0.90[2] (27)	p=0.17[2] (13)	p=0.40[2] (5)	p=0.40[2] (10)
**AEDs (No)**	p=0.42[1] (27)	p=0.97[1] (13)	p=0.87[1] (5)	p=0.92[1] (10)

Supplementary Table 2: Correlation analyses between postictal IL-6 and ICAM-1 levels and verified seizure-related parameters in TLE patients during videoEEG monitoring.

Data presented as p-value with the number of patients included in each analysis (n). [1]= Spearman correlation, [2]= Mann-Whitney U-test. p-values ≤0.05 were considered statistically significant. No = number.

TLE	IL-6 (n) 6hrs	INF-y (n) 6hrs	Mip1b (n) 6hrs	MDC (n) 6hrs	TARC (n) 6hrs	ICAM-1 (n) 24hrs
**Correlation analysis to ratio of postictal / interictal protein levels in TLE**						
**Index seizure duration (s)**	p=0.99[1]	p=0.10[1] (11)	p=0.06[1] (13)	p=0.70[1] (13)	p=0.72[1] (13)	p=0.40[1] (13)
**Index seizure with bilateral convulsions (yes/no)**	p=0.09[2] (13)	p=0.33[2] (11)	p=0.56[2] (13)	p=0.13[2] (13)	p=0.52[2] (13)	p=0.47[2] (13)
**No of seizures between index and blood sampling**	p=0.76[1] (13)	p=0.98[1] (11)	p=0.13[1] (13)	p=0.99[1] (13)	p=0.93[1] (13)	p=0.29[1] (13)
**No of bilateral convulsions between index and blood sampling**	p=1.0[2] (13)	p=0.38[2] (13)	**p=0.034**[2] (13)	p=0.33[2] (13)	p=0.20[2] (13)	*N.D*
**Time between last seizure and postictal blood sampling (h)**	p=0.92[1] (13)	p=0.98[1] (11)	p=0.13[1] (13)	p=0.99[1] (13)	p=0.93[1] (13)	p=0.09[1] (13)
**Interictal activity (graded 0-3)**	p=0.78[1] (13)	p=0.098[1] (11)	p=0.46[1] (13)	p=0.25[1] (13)	p=0.60[1] (13)	0.87[1] (13)
