# Supplementary material for: Immune response in blood before and after epileptic and psychogenic non-epileptic seizures
Source: Heliyon. 2023 Feb 21;9(3):e13938. doi: 10.1016/j.heliyon.2023.e13938 (PMC9988551; doi:10.1016/j.heliyon.2023.e13938)
Supplement: Questionnaire_Research project participants_English [file mmc3.pdf]

Questionnaire – English translation

Questionnaire for research project participant at admittance to video-EEG monitoring

1. When was your last seizure?

2. How many seizures per week have you experienced in the last six months, and the last month, respectively?

3. Which symptoms do you experience during your seizures? Could you describe what one of your typical seizures feels like, to you?

4. How much physical exercise do you do per week (pulse above 100 bpm for a duration of >30 min)? Circle your answer:

   None 1-3 times/week >3 times/week

5. How much alcohol did you consume in the last month? Circle your answer:

Alternative 1: < 1 glass of wine or 1 strong beer/week

Alternative 2: In between alternatives 1 and 3.

Alternative 3: Women: > 2 bottles of wine or 10 bottles of strong beer/week, Men: >3 bottles of wine or 15 bottles of strong beer/week

6. Have you hit your head so badly you fainted, within the last six months?

7. Have you undergone brain surgery? If YES, when?

8. Do you have a current inflammatory disease, such as inflammation in nerves or joints? If so, which one?

9. Do you have a current neurological or psychiatric disorder? If so, which one?

---

10. Do you have any hereditary disorders in your family? Do you have a hereditary disorder?

---

11. Do you have a diagnosed sleep disorder?

---

12. Do you have a diagnosed neuropsychiatric disorder, e. g. autism?

---

13. Have you received ECT(Electroconvulsive Therapy) within the last six months?

---

14. Are you pregnant?

---

15. Which medications have you been taking regularly in the last month?

Date:____________________ Location:________________________
